# Relative position of the central hole after EVO-ICL implantation for moderate to high myopia

**DOI:** 10.1186/s12886-020-01569-9

**Published:** 2020-07-28

**Authors:** Xiaojian He, Lingling Niu, Huamao Miao, Feng Zhao, Xingtao Zhou

**Affiliations:** 1 Department of Ophthalmology, Hangzhou First People’s Hospital, Zhejiang University School of Medicine, Hangzhou, China; 2 Eye Institute and Department of Ophthalmology, Eye & ENT Hospital, Fudan University, Shanghai, China; 3 NHC Key Laboratory of Myopia (Fudan University); Key Laboratory of Myopia, Chinese Academy of Medical Sciences, Shanghai, China; 4 Shanghai Research Center of Ophthalmology and Optometry, Shanghai, China; 5 Shuguang Hospital affiliated to Shanghai University of Traditional Chinese Medicine, Shanghai, China

## Abstract

**Background:**

This study aimed to evaluate the relative position of the central hole (C_H_) of EVO Implantable Collamer Lens (EVO-ICL), the pupil center (C_P_), and the corneal center (C_C_) after implantation of EVO-ICLs for moderate to high myopia.

**Methods:**

Eighty-nine eyes of forty-seven patients with moderate to high myopia were evaluated. The mean preoperative spherical equivalent (SE) was − 12.58 ± 4.13D. A routine postoperative follow-up was performed within 1 ~ 12 months. Positions of the C_H_ of EVO-ICLs, the C_P_ and the C_C_ were recorded using a slit lamp anterior segment photography system, and their relative distances were calculated with the Visio image analysis software.

**Results:**

All surgeries were performed safely, and no complications were observed in follow-ups 4.3 ± 4.82 months after surgery. At the last follow-up, the safety index (postoperative CDVA/preoperative CDVA) was 1.23 ± 0.48, and the efficacy index (postoperative UDVA/preoperative CDVA) was 1.08 ± 0.31. The C_H_ in 85 eyes (95.51%) was superior to the C_C_, with 47.19% (42/89) on the temporal side and 48.31% (43/89) on the nasal side. The C_H_ in 84 eyes (94.38%) was located on the temporal side of the C_P_, with 56.18% (50/89) superior and 38.2% (34/89) inferior to the C_P_. The C_P_ of 85 eyes (95.51%) was superior on the nasal side of the C_C_. On the defined x-axis, the average distance from the C_H_ to C_C_ was significantly shorter than the average distance from the C_P_ to C_C_ (*p* < 0.001).

**Conclusions:**

An imperfect match between the central hole of EVO-ICLs and the pupil center does not necessarily indicate ICL dislocation. Compared to the pupil center, the position of the central hole of EVO-ICL is closer to the corneal center.

## Background

Implantation of a posterior chamber phakic Implantable Collamer Lens (ICL™, STAAR Surgical, Nidau, Switzerland) has proved to be safe and effective to correct moderate to high myopia [1, 2]. There is no limit to the corneal thickness of the ICL implantation, which preserves the integrity of the cornea and the accommodation function of the lens after surgery. More importantly, the implantation of ICL is reversible.

In refractive surgery, accurate centration helps to maximize visual outcomes and is considered to be of great importance [3]. Ideally, the position of the corrected center should be aligned to the optical axis or to the intersection point between the optical axis and the anterior corneal surface [4]. However, it is not possible to directly observe the central position of ICL. EVO Implantable Collamer Lens (EVO-ICL) with CentraFLOW technology is characterized by a 360 μm central hole that can allow natural circulation of the aqueous humour and can decrease the risk of cataract, high intraocular pressure (IOP) and corneal endothelial cell loss after ICL implantation [5, 6]. Moreover, it provides an opportunity to directly observe the central position of the EVO-ICL in the posterior chamber. However, it is a common occurrence that the location of the central hole (C_H_) of the EVO-ICL is not perfectly aligned with the pupil center (C_P_). As the structure of the anterior segment of the eye has changed after the implantation of ICL, ultrasound biomicroscopy (UBM) [7], optical coherence tomography (OCT) [8], and rotating Scheimpflug imaging (Pentacam) [9] are used to evaluate the position and the vault of the implanted ICL, as well as relationships between the ICL and its adjacent structures. However, the position of the ICL relative to the corneal center (C_C_), which was defined as the intersection between the longest horizontal and vertical diameters of the corneal ellipse, has not yet been reported. Therefore, in this study, we aimed to measure the position of the central hole of the EVO-ICL relative to the corneal center and the pupil center, which may be of potential clinical benefit.

## Methods

### Subjects

This observational study was conducted on patients who underwent EVO-ICL implantation from January 2016 to October 2016 in the Department of Eye & ENT Hospital of Fudan University. In accordance with the Declaration of Helsinki, all patients provided written informed consent after receiving detailed explanation of the risks and benefits of the study. The study was approved by the Ethics Committee of the Eye & ENT Hospital (EENT) Fudan University.

The study was conducted on 89 eyes from 47 patients (19 males and 28 females, mean age 29.6 ± 7.42 years) who were recruited. The inclusion criteria for patients who underwent ICL implantation were as follows: age between 20 and 45 years, stable refractive error (refractive error change of ≤0.50 D in the past 2 years), spherical refraction of over − 3.00 D, astigmatism of up to − 6.00 D, and no contact lens use for 2 weeks. Exclusion criteria were anterior chamber depth (ACD) < 2.80 mm, endothelial cell density (ECD) < 2000 cells/mm^2^, a history of ocular surgery, any chronic systemic disease, inflammation or trauma, and a history of ocular conditions other than myopia with or without astigmatism (suspicion of cornea, cataract, keratectasia, glaucoma, macular degeneration, retinal detachment, or neuro-ophthalmic disease).

### EVO-ICL

EVO-ICL corrects − 0.50 D to − 18.00D myopic spherical refraction and up to − 6.00 D cylindrical refraction. The size of the EVO ICL was individually selected based on the horizontal white-to-white (WTW) distance by IOLmaster and the ACD by Pentacam camera system (Oculus, Germany) following the manufacturer’s recommendations. We calculated the size on the website at http://ocos.staarag.ch. The software is version 4.08 for ICL V4c. Power calculation for the EVO-ICL was performed by the software provided by the manufacturer (STAAR Surgical) using a modified vertex formula.

### Surgical procedure and follow-ups

Two experienced surgeons (XZ and XW) performed all implantations. Chen X et al. have previously described the surgical procedure [9]. Briefly, before surgery, the pupils were dilated. The anterior chamber was filled with sodium hyaluronate (ProVisc, Alcon), and an EVO-ICL was inserted through a 3.0 mm incision in the temporal corneal limbus using an injector cartridge (STAAR Surgical Co), then positioned in the posterior chamber. The remaining viscoelastic surgical agent was removed using balanced salt solution, before a miotic agent was instilled into the anterior chamber. Postoperative medications included 0.3% tobramycin, 0.1% fluorometholone, non-steroidal anti-inflammatory (NSAID) and artificial tears eye drops.

We determined manifest refraction (spherical equivalent, SE), uncorrected distance visual acuity (UDVA), corrected distance visual acuity (CDVA), IOP (Canon, Japan), endothelial cell density (ECD, by SP-2000P, Topcon Corporation, Japan), standard slit-lamp biomicroscopic and funduscopic examinations preoperatively and all follow-ups. Anterior chamber depth (ACD) was measured from the corneal endothelium to the anterior lens using a Pentacam camera system and horizontal corneal diameter (white-to-white, WTW) using IOLMaster before surgery. Central corneal thickness (Pentacam), axial length (IOLMaster Carl Zeiss, Germany) and ultrasound biomicroscopy (UBM; Quantel Medical, France) were performed preoperatively.

### Image analysis

**Fig. 1:**
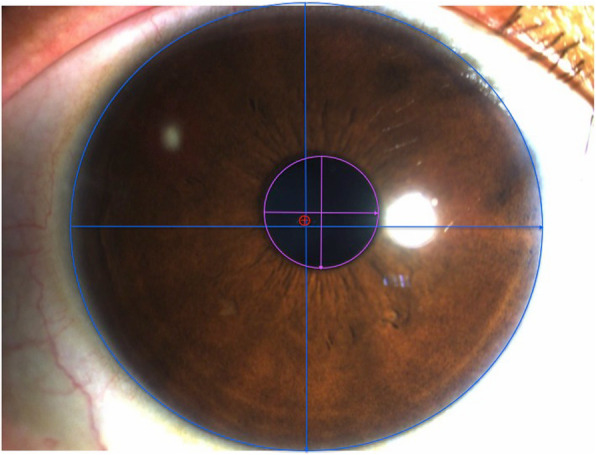
The red line and circle is shown for the central hole of the EVO-ICL, the purple line and circle is shown for the pupil center, and the blue line and circle is the corneal center of the right eye. Visio image analysis software was used for image analysis

Under the same illumination conditions of 330 lx, which was monitored using a photometer (Digitales Luxmeter, Sensorshop24^@^, Germany), all images were collected in the same room at a 10x magnification using a slit lamp anterior segment photographic system (YZ5FI Slit Lamp + SLICPS 2). Patients sat comfortably with their eyes facing straight ahead. The diffused light from the slit lamp was illuminated 60° from the temporal side. Using Visio image analysis software (version 2013), the size of the center hole was fixed to 360 μm (7.5 pixels) in both the horizontal (X-axis) and vertical (Y-axis) axis, and the image size was then adjusted accordingly. The average horizontal and vertical relative corneal diameters were 214.23 ± 14.66 pixels and 201.78 ± 12.39 pixels, respectively, and the average horizontal and vertical pupil diameters were 52.87 ± 11.95 pixels and 50.58 ± 11.65 pixels, respectively. Images confirmed that both the cornea and the pupil were elliptical (Fig. 1). The actual magnification of the image was calculated according to the following equation: WTW (mm)/relative horizontal diameter of the cornea (mm). Similar to the method reported by Hoang et al. [10], the central hole of EVO-ICL (C_H_), the corneal center (C_C_), and the pupil center (C_P_) were defined as the intersection between the longest horizontal and vertical diameters of the respective ellipse. Each measurement was performed three times by the same physician, and the results were averaged.

**Fig. 2:**
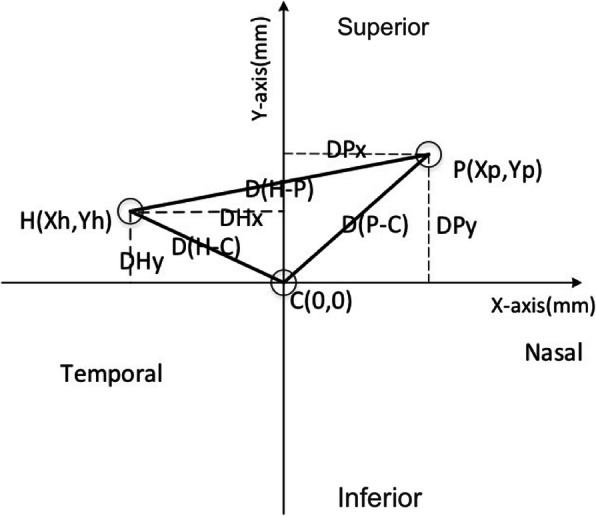
Relative position of the central hole of EVO-ICL, the corneal center and the pupil center

In this study, we set the C_C_ of the fitted corneal image as the reference point (0,0) and the position of C_H_ and C_P_ as (X, Y) using the following formula: D = (X^2^ + Y^2^)^1/2^ and calculated the relative distances from the center hole to the corneal center, D(H-C); the pupil center to the corneal center, D(P-C); and the center hole to the pupil center D(H-P) (Fig. 2). According to the equation: actual distance Ð = relative distance D * WTW (mm)/relative horizontal diameter of the cornea (mm), the actual distances of Ð(H-C), Ð(P-C) and Ð(H-P) were calculated respectively. In all images, the X-axis value of the left eye was converted to a negative value, which was converted to the same nasal and temporal direction as the right eye. The X-axis (ÐHx and ÐPx) and the Y-axis (ÐHy and ÐPy) on Ð(H-C) and Ð(P-C) are shown in Fig. 2. The X-axis (ÐHPx) and the Y-axis (ÐHPy) on Ð(H-P) were defined as the X-axis and the Y-axis of the vector of Ð(H-P).

### Kappa angle

Pupil offset was used as the estimate of the Kappa angle and defined as the vector between the corneal vertex and the pupil center in the corneal plane. Pupil offset was measured using Pentacam. The x-offset (K-x) and y-offset (K-y) of the vector were recorded respectively. A positive value is defined as the pupil center nasal to the pachy apex (x-offset) and as the pupil center upper to the pachy apex (y-offset).

### Statistical analysis

All statistical analyses were performed using Stata statistical software, version 14.1 (Stata Corporation, College Station, TX, USA). The Wilcoxon Signed-rank test was used for statistical analysis to compare Ð(H-C) and Ð(P-C), ÐHx and ÐPx, ÐHy, and ÐPy. Spearman’s correlation analysis was used to assess the correlation between Ð(H-C) and Ð(P-C), ÐHx and ÐPx, and ÐHy and ÐPy, K-x (K-y) and ÐHx (ÐHy), K-x (K-y) and ÐHPx (ÐHPy). Results are expressed as mean ± standard deviation (SD), and statistical significance was set at *p* < 0.05.

## Results

### Safety and efficacy

All surgical procedures were completed successfully, and no complications occurred throughout the entire follow-up period. The median follow-up period was 4.3 ± 4.82 months (range: 1 ~ 12 months). No postoperative complications were observed, and no loss of corrected distance visual acuity (CDVA) was recorded throughout the entire follow-up period. The mean preoperative spherical equivalent (SE) was − 12.58 ± 4.13 D (range: − 5.75 to − 22.75D). The safety index (postoperative CDVA/preoperative CDVA) was 1.23 ± 0.48. No patient had CDVA loss at any follow-up. The efficacy index (postoperative UDVA /preoperative CDVA) was 1.08 ± 0.31, and the mean vault was 525.93 ± 228.43 μm (range: 100 ~ 1070 μm). No eye of ECD decreased to < 2000 cells/mm^2^ throughout the entire follow-up period.

### Relative position of C_H_ to the C_C_ and the C_P_

**Fig. 3:**
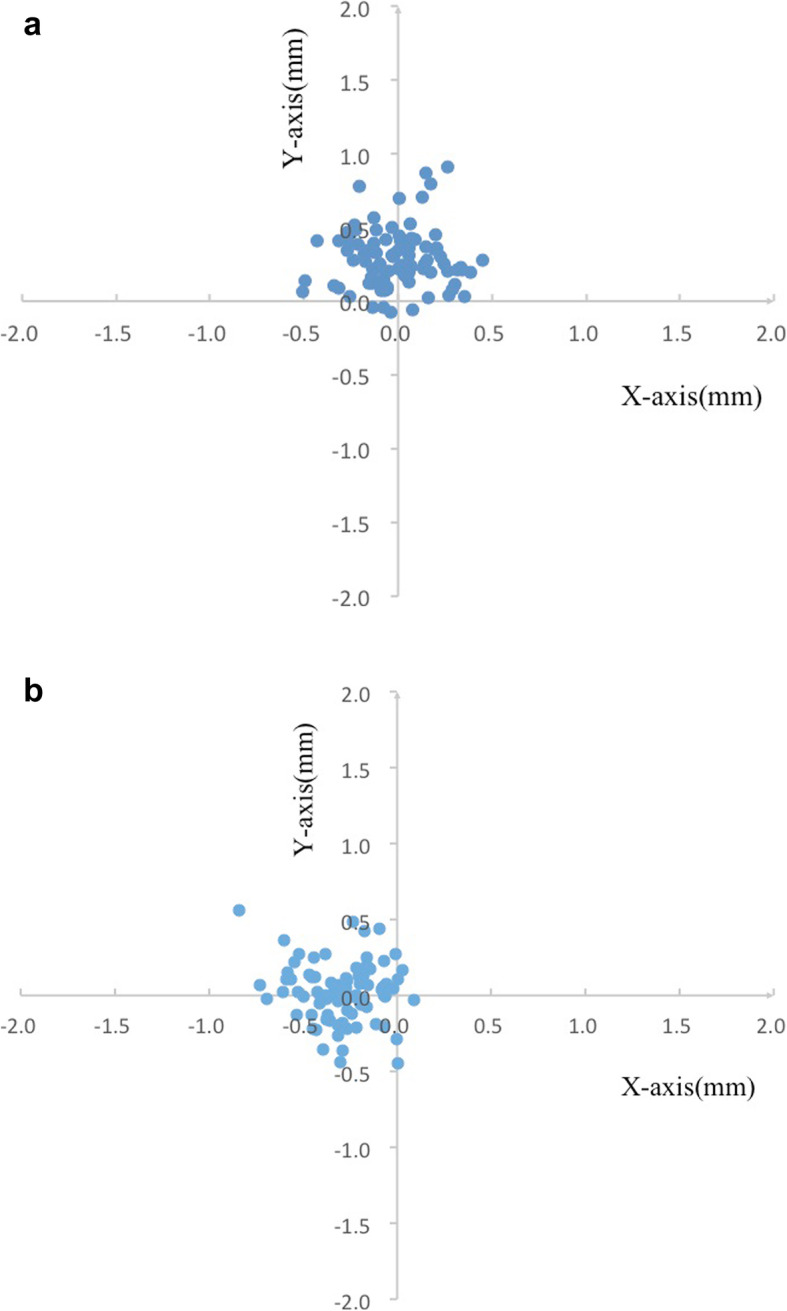
**a** Scatter plot of the central hole relative to the corneal center. (0, 0) represents the corneal center. **b** Scatter plot of the central hole relative to the pupil center. (0, 0) represents the pupil center

Relative to the C_C_, the C_H_ of EVO-ICL was superior to the C_C_ in 85/89 eyes (95.51%), with 42/89 (47.19%) superior on the temporal side, 43/89 (48.31%) superior on the nasal side, 3/89 (3.37%) inferior on the temporal side, and 1/89 (1.12%) inferior on the nasal side (Fig. 3a). Relative to the C_P_, the C_H_ was located on the temporal side in 84/89 eyes (94.38%), with 50/89 (56.18%) superior on the temporal side, 34/89 (38.2%) inferior on the temporal side, 2/89 (2.25%) superior on the nasal side, and 3/89 (3.37%) inferior on the nasal side (Fig. 3b).

### Relative position of the C_P_ to the C_C_

**Fig. 4:**
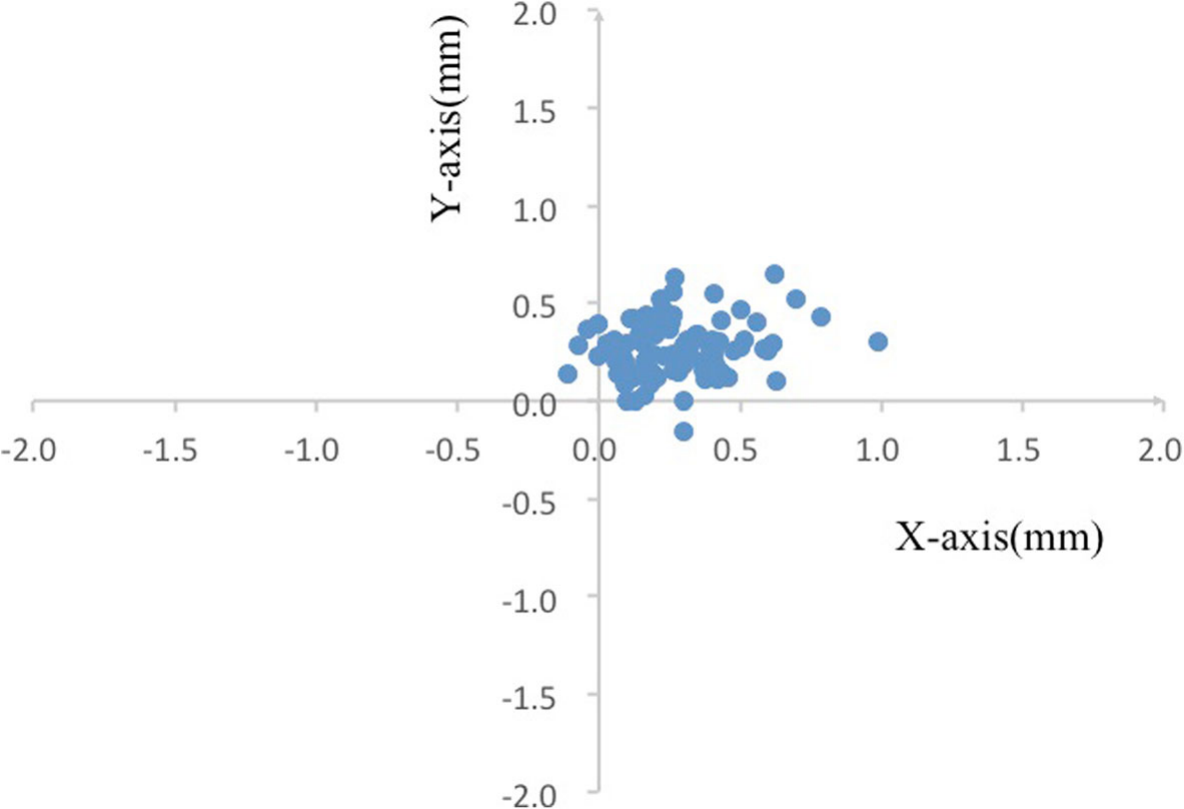
Scatter plot of the pupil center relative to the corneal center. (0, 0) represents the corneal center

C_P_ was superior on the nasal side of the C_C_ in 85/89 eyes (95.51%), with 3/89 (3.37%) superior on the temporal side, and 1/89 (1.12%) inferior on the nasal side in (Fig. 4).

### Distance between the C_H_, the C_C_ and the C_P_

The average Ð(H-C) and Ð(P-C) were 0.37 ± 0.18 mm and 0.42 ± 0.18 mm respectively, and were approaching a statistically significant difference (*p* = 0.058). The average ÐHx and ÐPx were 0.16 ± 0.12 mm and 0.28 ± 0.19 mm respectively, and were statistically different (*p* < 0.001). The average ÐHy and ÐPy were 0.30 ± 0.19 mm and 0.27 ± 0.14 mm respectively, and the difference was not statistically significant (*p* = 0.624). The average Ð(H-P) was 0.35 ± 0.18 mm (range: 0.05 ~ 1.01 mm). Though there was no significant correlation between Ð(H-C) and Ð(P-C) (*r* = 0.20, *p* = 0.062), ÐHx was positively correlated with ÐPx (*r* = 0.57, *p* < 0.001) and ÐHy with ÐPy (*r* = 0.42, *p* < 0.001).

### Relationships of kappa angle with Ð(H-C) and Ð(H-P)

There was no significant correlation between K-x and ÐHx (*r* = − 0.011, *P* = 0.923), or between K-y and ÐHy (*r* = − 0.155, *P* = 0.162), while the relationships between K-x and ÐHPx (*r* = − 0.253, *P* = 0.021), K-y and ÐHPy (*r* = − 0.435, *P* < 0.001) was significantly correlated postoperatively.

## Discussion

The new EVO-ICL has reduced risk of high intraocular pressure (IOP), cataracts, and endothelial cell loss after ICL implantation and is more effective and safer than conventional ICLs due to natural flow through a central whole [5, 6, 11]. The position of the ICLis critical during follow-up since it may be of consequence to corrected visual acuity as well as postoperative complications such as cataracts and glaucoma [5, 11]. However, the position of conventional ICLs, such as the V4, is difficult to evaluate without the use of UBM or a Pentacam [12, 13]. In this study, we were able to evaluate the relative position of the EVO-ICL in the posterior chamber directly based on the position of the center hole in a slit lamp image, without the need for additional inspection.

The selection of ICL diameter depends mainly on the preoperative horizontal corneal diameter (WTW) and anterior chamber depth [14]. There are many studies available on the correlation between WTW and STS. Seo et al. [15] reported that the WTW diameter matched with the distance of the STS on the horizontal direction as measured by UBM. However, Biermann [16] compared emmetropic and myopic eyes, and found that the horizontal direction of the emmetropic STS had a significant correlation with WTW, but there was only a weak correlation between the two in the myopic population. Though the STS distance may not completely be estimated by WTW, the vertical ciliary sulcus was longer than the horizontal ciliary sulcus, which meant the ciliary sulcus was vertical ellipse had been identified. ICLs are usually positioned horizontally in the ciliary sulcus with a rotation of< 5° even implantations of toric intraocular collamer lenses (TICLs) [17, 18]. The haptics of ICL are located at the ciliary sulcus, Hence, the horizontal position of the central hole may be closer to the horizontal center of the ellipse ciliary sulcus. Here, we described the relative position of the central hole, the corneal center and the pupil center, and found that most of central holes of EVO-ICLs were superior to the corneal center and were on the temporal side of the pupil center. Although the difference between Ð(H-C) and Ð(P-C) did not reach statistical significance difference (*p* = 0.058), Ð(H-C) was significantly shorter than Ð(P-C) on the X-axis (*p* < 0.001), indicating that the center of the ICL should be positioned relative to the corneal center, which is consistent with our observation. Therefore, it is reasonable to evaluate the position of the EVO-ICL according to the relative position of the central hole to the corneal center.

The mean distance between the pupil center and the corneal center in our study was 0.42 ± 0.18 mm, which was longer than the 0.27 ± 0.14 reported by Medby et al. [19]. This discrepancy could possibly have resulted from the different imaging methods or due the different research conditions. It has been reported that the location of the pupil center may change under various illumination conditions [20–22]. Yang et al. [20] reported that the pupil center moved temporally towards the corneal center under mesopic or pharmacologically dilated conditions. And Fay [23] reported that, as the pupil constricts, the most common shift of the pupil center was in a superonasal direction. We also found that the central hole and the pupil center were both upwards relative to the corneal center. Other studies using UBM have shown that ICLs were in contact with the posterior surface of iris [7, 24], suggesting that the mechanical friction between the iris and the ICL could be responsible for the concordant movement of the implanted ICLfollowing the shift of the pupil. Spearman’s correlation analysis showed that ÐHx was positively correlated with ÐPx (*r* = 0.57, *p* < 0.001), and ÐHy was positively correlated with ÐPy (*r* = 0.42, *p* < 0.001), suggesting that shifts of both the central hole of the EVO-ICL and the pupil center relative to the corneal center were concordant in the horizontal and vertical directions. However, there was no direct correlation between Ð(H-C) and Ð(P-C) (*r* = 0.20, *p* = 0.062).

Consideration of the kappa angle is important for correct centration of refractive treatments [25], therefore the influence of kappa angle on the position of ICL was analyzed. We can find that the distance between K-x and ÐHPx or K-y and ÐHPy was significantly corrected, while not K-x and ÐPx or K-y and ÐPy. This may indicate that due to the influence of the Kappa angle, if the pupil center is used as the reference, the position of the central hole would affected by the position and diameter of the pupil. In that case the results may be different under different lighting environments. However if the corneal center is used as the reference, the results may be relatively stable. In this study, we used the slit lamp anterior segment photographic system and objective image analysis to evaluate the relationship between the central hole and the corneal center. This has the benefits of being easy to use, non-invasive, and not requiring any additional equipment, which means that it has certain feasible advantages over other methods for clinical observation.

The current study has several limits. Firstly, this was a cross-sectional study with inconsistent follow-up time from 1 month to 12 months after surgery, and it lacked a comparison of the position changes over time, especially in the early period after surgery. As the vault decreased most significantly at first months after surgery [26], it would have been useful to determine if the center of EVO changed from the first day to the first-month visit, which may influence the position of ICL. Secondly, we did not analyze the relationships between the distance of the central hole to the corneal center and clinical outcomes, such as visual quality, after ICL implantation which may need to be further investigated in future studies.

## Conclusions

Although the central hole of the EVO-ICL does not perfectly match the pupil center, it is not necessarily indicative of an ICL dislocation. Compared to the pupil center, the position of the central hole of the EVO-ICL is closer to the corneal center.

## Data Availability

Data and materials are available upon request from the corresponding author at doctzhouxingtao@163.com.

## Abbreviations

EVO-ICL: Evolution Visian Implantable Collamer Lens
SE: Spherical equivalent
D: Diopter
C_H_: Central hole of EVO-ICL
C_C_: Corneal center
C_P_: Pupil center
IOP: Intraocular pressure
UBM: Ultrasound Biomicroscopy
OCT: Optical coherence tomography
ACD: Anterior chamber depth
ECD: Endothelial cell density
WTW: White-to-white
NSAID: Non-steroidal anti-inflammatory drugs
UDVA: Uncorrected distance visual acuity
CDVA: Corrected distance visual acuity
SD: Standard deviation
STS: Sulcus to sulcus
